# Exploratory Analysis of the Microbiological Potential for Efficient Utilization of Fiber Between Lantang and Duroc Pigs

**DOI:** 10.3389/fmicb.2018.01342

**Published:** 2018-06-22

**Authors:** Penghui Cheng, Yan Wang, Juanboo Liang, Yinbao Wu, Andredenis Wright, Xindi Liao

**Affiliations:** ^1^College of Animal Science, South China Agricultural University, Guangzhou, China; ^2^Institute of Tropical Agriculture and Food Security, Universiti Putra Malaysia, Seri Kembangan, Malaysia; ^3^School of Animal and Comparative Biomedical Sciences, College of Agriculture and Life Sciences, University of Arizona, Tucson, AZ, United States

**Keywords:** pig, metagenome, dietary fiber, microbiome, breed

## Abstract

There is growing interest in the use of unconventional feed ingredients containing higher dietary fiber for pig production due to increasing prices of cereal grains and the potential health benefits of dietary fiber on host animals. This study aimed to gain insight into the community-wide microbiome population between the Chinese native Lantang pigs and the commercial Duroc pigs to uncover the microbiological mechanisms for the degradation capacity of fiber in pigs. Utilizing the metagenomics approach, we compared the phylogeny and functional capacity of the fecal microbiome from approximately 150-day-old female Lantang and Duroc pigs fed a similar diet. The structure of the fecal microbial community from the two pig breeds was different at the genus level; the number of genes associated with fiber degradation was higher in Lantang pigs. Further analysis and prediction of their functions from the fecal microbiomes of the two pig breeds revealed that the degradation capacities of fiber, branched chain fatty acids, and oligosaccharides were higher in Lantang pigs. The ability of lignocellulose bonding modules and the transport capacities of xylose, L-arabinose, ribose and methyl galactose were also higher in Lantang pigs. Similarly, the metabolic capacities of xylose, ribose, and fucose and the potential effectiveness of the tricarboxylic acid cycle (TCA) and gene abundance in the hydrogen sink pathway were higher in the fecal microbiome from Lantang pigs. Lantang pigs have a higher capacity to utilize dietary fiber than Duroc pigs, and the differences in the capability to utilize dietary fiber between the indigenous and commercial pigs could be differences in the composition and biological function of the gut microbiota.

## Introduction

Starch and fats are the traditional sources of dietary energy in commercial pig feeding programs. Conversely, dietary fiber, because of its negative impacts on energy digestibility and restrictions on feed intake, is usually maintained at the lowest concentration possible (Noblet and Le Goff, 2001; Wenk, 2001; Zijlstra et al., 2010). There is, however, a growing need to use alternative feed ingredients with relatively higher concentrations of dietary fiber because prices of cereal grains have increased due to other usages, including for biofuel production (Stein and Shurson, 2009). A better understanding of the efficient utilization of dietary fiber may provide greater opportunities to successfully introduce alternative feed ingredients with higher fiber into pig diets.

To mitigate the negative impacts of fibrous ingredients on the diets of growing pigs, the focus of research was aimed toward maximizing the beneficial effects of fiber, which is largely derived from its fermentability in the hind gut of pigs. Several previous studies have shown that native pig breeds, such as Mong Cai (Vietnam), Kune-Kune (New Zealand), Schwaebisch Haellisches (Germany) and Bunte Bentheimer (Germany), have a greater capacity for digestion of dietary fiber than commercial crossbred pigs (Morel et al., 2006; Len et al., 2007; von Heimendahl et al., 2010). Dietary fiber is not digestible in the small intestine but is fermentable in the large intestine due to the diverse community of microorganisms harboring the large intestine. The higher digestibility of dietary fiber in native pig breeds was previously suggested to be due to physiological and anatomical differences, including a slower rate of passage of digesta in the gastrointestinal tract (GIT)and the length and volume of the large intestine (Grieshop et al., 2001; Yen et al., 2004). Other researchers have reported that the host genotype is involved in the selection of a host-specific set of intestinal bacteria and that differences in the composition of the intestinal microbiota exist among different breed pigs (Friswell et al., 2010; Luo et al., 2012; Hildebrand et al., 2013; Pajarillo et al., 2015). The above findings thus suggest that differences in the capability to utilize dietary fiber between indigenous and commercial pigs are due to differences in the compositions of their gut microbiota.

Next-generation sequencing technologies have been used to characterize the diversity and functional capacity of a range of microbial communities in the GIT of pigs (Lamendella et al., 2011; Looft et al., 2012; Xiao et al., 2016). However, to the best of our knowledge, this study was the first to co-compare the function of the fecal microbiomes from native Chinese Lantang and commercial Duroc pigs fed a similar diet to elucidate the potential mechanisms responsible for the differences in fiber utilization between the two pig breeds.

## Materials and Methods

### Animals and Fecal Sample Collection

This study was conducted according to the protocols approved by the South China Agricultural University’s Animal Care and Use Committee. Fecal samples were collected from three approximately 5-month-old female Lantang and Duroc pigs from a commercial pig farm (Guangdong Bangling, southern China). The pigs were individually penned and fed the same diet without antibiotics from weaning (4 weeks old) to 150 days old. The diet, containing 61% corn, 22% soybean meal, 13% wheat bran and 4% premix, was offered (approximately 1 kg/portion) twice daily (0800 and 1600 h). Fecal samples (100–200 g) were manually collected directly from the rectum within 1 h after feeding from three pigs in each breed and immediately stored in CO_2_ pre-flushed plastic containers, placed on crushed ice, and transported to the laboratory within 4 h after collection.

### DNA Extraction and Metagenomic Sequencing

Genomic DNA was extracted from the fecal samples using the QIAamp DNA Stool Mini Kit (Qiagen, Valencia, CA, United States) following the manufacturer’s instructions (0.25 g of each fecal sample). DNA quality and quantity were determined with a NanoDrop (ND-1000) spectrophotometer (NanoDrop Technologies, Wilmington, DE, United States). DNA samples were stored at -20°C until use. Two DNA pools were constructed with equal amount of fecal DNA from each breed, including one DNA pool from 3 Lantang pigs and the other DNA pool from 3 Duroc pigs. DNA libraries for metagenomic sequencing were constructed following the manufacturer’s instruction (Majorbio Co., Ltd). Paired-end library with insert size of ∼350 base pairs of each DNA pool was constructed and sent for metagenomics sequencing on a Hiseq 2000 platform (Illumina, United States). Preliminary analysis of the data involved removing reads with an average quality score lower than Q20 (1% error rate) and low-quality bases larger than 20% of the total read length using the NGS QC Toolkit (Patel and Jain, 2012). Sequence reads were then compared to *Sus scrofa* genomic sequences using the BLASTN program, and the reads corresponding to pig genome DNA were removed for downstream analysis (Fang et al., 2012). An average of 0.71% of the reads corresponding to pig genome sequences were removed (**Table 1**). The sequence reads that remains after culling of low quality reads and removal of host contaminated reads were referred as high-quality reads hereon. All gene sequencing data were submitted to the European Nucleotide Archive(ENA)with the accession numbers ERP107787.

**Table 1:** Summary of metagenome database.

Parameter	DR1	DR2	DR3	LT1	LT2	LT3
No. of raw reads	60,063,352	66,594,482	62,369,116	62,828,798	63,142,492	65,190,166
Total sequence length (bp)	6,066,398,552	6,726,042,682	6,299,280,716	6,345,708,598	6,377,391,692	6,584,206,766
No. of high quality reads	50,074,840	56,552,948	52,359,526	53,439,566	52,667,206	54,723,672
% of high quality reads	83.37%	84.92%	83.95%	85.06%	83.41%	83.94%
% of pig genome sequences	1.23%	0.01%	0.18%	0.19%	2.49%	0.14%
Mean GC percent	42%	42%	42%	43%	43%	43%
N50 (bp)	289	281	255	335	325	352
Largest contig (bp)	228,926	74,215	194,266	77,943	102,297	112,394
Reads in contigs	12,674,097	12,724,449	9,437,369	13,798,295	15,357,664	14,249,094
Identified protein reads						
Reads with KEGG information	12,680,214	16,250,830	13,158,106	15,431,278	13,098,396	15,537,712
Reads with CAZy information	2,504	3,089	2,165	3,752	3,450	4,096

### Sequences Assembly and Open Reading Frames (ORF) Prediction

The high quality sequences from each sample were assembled using meta-velvet software (Namiki et al., 2012). FragGeneScan (Rho et al., 2010) software was used to predict ORFs in contigs length larger than 1000 bp for each sample.

### Bioinformatics and Statistical Analysis

The DNA sequences were compared against the National Center for Biotechnology Information non-redundant protein database (NCBI-nr) using BLASTx (bit score≥40). The taxonomic information of the protein-coding gene sequences was obtained by parsing the NCBI-nr comparison results using the lowest common ancestor algorithm in MEGAN with default parameters (Huson et al., 2007). Sequences assigned to bacteria and archaea were downloaded for further analysis. The relative abundance of a given taxon was calculated as the percentage of the number of sequences assigned to the taxon divided by the total number of sequences assigned to all taxa.

Functional information of these sequences was first obtained from matching genes in the NCBI-nr database. To further reveal the function of these genes, the protein-coding sequences were assigned to the Kyoto Encyclopedia of Genes and Genomes (KEGG) database using BLASTx with a threshold of bit scores ≥40. The top blast hit was used as annotation before the sequences were assigned to KEGG pathways. The maximum *e*-value of 1e^-5^, a minimum percent identity of 60, and a minimum alignment length of 30 were applied as the parameter settings. Lastly, annotations based on the carbohydrate-active enzymes database (CAZy) were provided for all reads. Sequences were analyzed with the CAZyme Analysis Toolkit (CAT) using Pfam-based annotation with an *e*-value of e^-5^ (Park et al., 2010).

The difference of taxonomic abundances and microbiome CAZy database of the fecal microbiomes from the two pig breeds pig were compared by Lefse (Linear discriminant analysis effect size) analysis (Segata et al., 2011). we first use the non-parametric factorial Kruskal–Wallis sum-rank test to detect features with significant differential abundance, Lefse uses linear discriminant analysis (LDA) to estimate the effect size of each differentially abundant feature. An alpha = 0.05 was used in Wilcoxon rank sum test, and the log value for the LDA analysis was set to be less than 2.0. *P*< 0.05 was considered statistically significant difference among groups. We selected the RPKG (reads per kilobase per genome equivalent) normalization method to calculate our KEGG databases before the multiple test correction analysis according to the previous report (Nayfach and Pollard, 2015). Statistical Analysis were performed using DEseq2 package for R (Love et al., 2014). Differences were considered significant at *P* < 0.05 for an adjusted *p*-value.

## Results

### Sequencing Output

Whole-community microbial DNA from pig fecal samples was sequenced. An average of 63,008,983 and 63,720,485 reads were generated from Duroc and Lantang pigs, respectively. In terms of sequence quality, an average score of Q20 was obtained for all samples (denoting the accuracy of a base call to be more than 99.2%). Approximately 0.53% of the total sequences were assigned to the pig (Sus scrofa domestica) genome. The reads obtained from Illumina sequencing were assembled to an average of 11,611,972 (Lantang) and 14,468,351 (Duroc) contigs, with the largest contig being 165,862 bases for the Duroc pig and 97,545 bases for the Lantang pig. Using KEGG and CAZy databases, the protein-coding genes averaged 14,029,717 and 2,586 for Duroc pigs, respectively, and 14,689,129 and 3,766 for Lantang pigs, respectively (**Table 1**).

### Phylogenetic Analysis of the Fecal Microbiome

**FIGURE 1:**
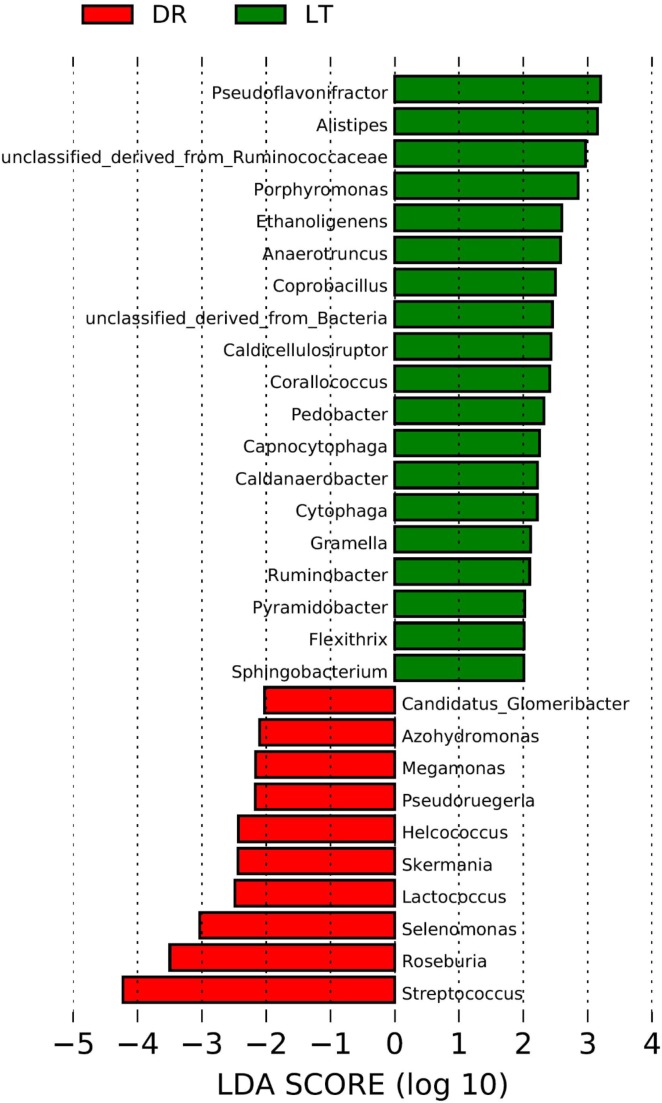
Difference in genera abundance in the fecal bacterial community from two pig breeds using Lefse software. The data analysis was performance using the Lefse software. Taxa enriched in the Drouc group (DR) are indicated with a negative LDA score (Red), and taxa enriched in the Lantang group (LT) have a positive score (Green). Only taxa meeting an LDA significant threshold of 2 are shown.

The rarefaction curves approached plateau (Supplementary Figure S1) illustrate that the sequence coverage of available phylotypes was adequate. According to the phylogenetic classification based on the NCBI-nr database, significant differences were found between the fecal microbiomes from the two pig breeds at the genus level (**Figure 1**). Based on Lefse, the genera *Pseudoflavonifractor, Alistipes, Porphyromonas, Ethanoligenens, Anaerotruncus, Coprobacillus, Caldicellulosiruptor, Corallococcus, Pedobacter, Capnocytophaga, Caldanaerobacter, Cytophaga, Gramella, Ruminobacter, Pyramidobacter, Flexithrix, Sphingobacterium*, and a genus of the family *Ruminococcaceae* were more abundant in the fecal microbiome from Lantang pigs relative to Duroc pigs. In contrast, bacteria belonging to the genera *Glomeribacter, Azohydromonas, Megamonas, Pseudoruegeria, Helcococcus, Skermania, Lactococcus, Selenomonas, Roseburia, Streptococcus* were more abundant in the fecal microbiome from Duroc pigs than in Lantang pigs.

### Overview of the Metabolic Potential of the Fecal Microbiome From the Two Pig Breeds

Functional metagenomic coverage of the fecal community of the two pig breeds was assessed by rarefaction analysis using protein-coding sequences (Supplementary Figure S2). As illustrated in Supplementary Figure S1, all rarefaction curves passed the steep region and leveled off to where fewer new functions were found, even after increasing the number of new sequences, thereby indicating acceptable coverage of dominant genomes of the fecal microbiome.

### Comparison of Complex Carbohydrate Utilization by the Fecal Microbiome From the Two Pig Breeds

To compare the differences in fiber utilization between the fecal microbiomes from the two pig breeds, modules that catalyze the breakdown and modification of carbohydrates were analyzed.

We found that glycoside hydrolase (GH), polysaccharide lyases, carbohydrate esterases and carbohydrate-binding modules were more abundant in the fecal microbiome of Lantang pigs than in Duroc pigs (*P* < 0.05). However, the number of genes involved in auxiliary activity did not differ between the two fecal microbiomes (*P* > 0.05) (**Table 2**).

**Table 2:** Module differences in the carbohydrate-active enzymes in the fecal microbial community from two pig breeds.

Item	Duroc	Lantang	*P*-value	*P.*adjust
Glycoside hydrolases	934.33 ± 91.87	1441.00 ± 91.00	0.017	0.050
Polysaccharide lyases	30.33 ± 6.89	50.33 ± 2.19	0.049	0.050
Carbohydrate esterases	285.67 ± 34.37	452.33 ± 31.71	0.024	0.050
Carbohydrate-binding modules	463.67 ± 38.58	637.00 ± 28.51	0.022	0.050
Auxiliary activity	25.67 ± 6.36	44.33 ± 5.04	0.083	0.150

Genes encoding plant cell wall-targeting GH proteins, retrieved from the microbiome metagenome from the Duroc and Lantang pigs, are presented in **Table 3**. The GH proteins were grouped according to their major functional role in plant fiber degradation. Comparative analysis of the repertoire of GH families recovered from the Duroc and Lantang fecal microbiomes revealed both similarities and differences. The gene numbers of debranching enzymes and oligosaccharide-degrading enzymes were higher in the fecal microbiome of Lantang pigs compared to Duroc pigs (*P* < 0.05). However, the gene numbers of cellulases and endo-hemicellulases in the two fecal microbiomes were similar. Within the fecal microbiomes of Duroc and Lantang pigs, enzymes belonging to the GH43, GH3, GH2, GH5, and GH28 families predomine (**Table 3**). The most abundant GH in the two pig breeds was the GH43 family of enzymes, acting mainly as β-xylosidases. The gene copy numbers of GH2 and GH3 enzyme families, which encompass β-galactosidases, β-glucosidases, and exoglucanases, as well as xylosidases are involved in the breakdown of a variety of oligosaccharides. These fore mention genes were significantly higher (*P* < 0.05) in the Lantang fecal microbiome than the Duroc fecal microbiome. GH5 endoglucanases are involved in cellulose breakdown and had the same gene copy number (*P* > 0.05) in both fecal microbiomes.

**Table 3:** Number of genes containing GH domains in the fecal microbiome from Duroc and Lantang pigs.

GH Category	Duroc	Lantang	*P*-value	*P*.adjust
**Cellulases**				
GH5	35.67 ± 4.98	46.00 ± 6.81	0.288	0.275
GH6	ND	ND		
GH7	ND	0.33		
GH9	7.00 ± 0.58	12.00 ± 1.53	0.038	0.049
GH44	ND	ND		
GH45	0.33 ± 0.33	0.33 ± 0.33	1.000	1.000
GH48	ND	ND	ND	
Total	43.00 ± 5.13	58.67 ± 7.54	0.161	0.127
**Endohemicellulases**				
GH8	12.33 ± 2.84	10.67 ± 1.45	0.630	0.827
GH10	17.00 ± 2.31	22.67 ± 4.84	0.350	0.375
GH11	0.67 ± 0.33	0.33 ± 0.33	0.519	0.456
GH12	0.33	ND		
GH26	12.33 ± 1.86	21.33 ± 3.28	0.075	0.127
GH28	19.00 ± 1.53	38.67 ± 6.89	0.049	0.050
GH53	14.67 ± 4.33	21.00 ± 0.58	0.221	0.376
Total	76.33 ± 11.86	114.67 ± 15.34	0.119	0.127
**Debranching enzymes**				
GH51	13.33 ± 2.03	19.33 ± 1.67	0.084	0.121
GH54	ND	0.33		
GH62	ND	ND		
GH67	3.67 ± 0.67	6.33 ± 0.33	0.023	0.043
GH78	12.33 ± 1.86	35.33 ± 1.20	0.0005	0.050
Total	29.33 ± 2.67	61.33 ± 1.45	0.0005	0.046
**Oligosaccharide-degrading enzymes**				
GH1	11.33 ± 2.85	13.00 ± 1.53	0.633	0.513
GH2	28.00 ± 4.62	55.67 ± 3.28	0.008	0.050
GH3	53.00 ± 7.64	76.33 ± 2.40	0.043	0.050
GH29	7.33 ± 0.88	30.33 ± 0.33	0.00001	0.046
GH35	4.67 ± 1.20	14.33 ± 3.18	0.047	0.050
GH38	2.33 ± 0.67	4.33 ± 0.67	0.101	0.099
GH39	3.00 ± 0.58	1.33 ± 0.67	0.132	0.105
GH42	2.67 ± 0.33	8.33 ± 1.33	0.015	0.043
GH43	76.00 ± 6.56	100.33 ± 11.17	0.133	0.127
GH52	ND	ND		
Total	188.33 ± 22.83	304.17 ± 29.14	0.018	0.050

*ND denotes data that were not detected.*

### Comparative Fecal Microbial Metagenomics Based on the KEGG Analysis

#### Bacterial Sugar Transporters in Lignocellulosic Hydrolysate Utilization

KEGG analysis showed that the abundance of sugar transporter genes in lignocellulosic hydrolysate utilization were clearly distinguishable in the fecal microbiomes of the two pig breeds. Compared to Duroc pigs, the numbers of arabinogalactan oligomer/maltooligosaccharide, lactose/L-arabinose, methyl-galactoside, d-xylose and ribose transporter genes in Lantang pigs were significantly higher (*P* < 0.05). However, the numbers of raffinose, stachyose, and melibiose transporter genes were significantly higher in Duroc pigs (*P* < 0.05) (Supplementary Figure S3 and Supplementary Table S1).

#### Bacterial Simple Sugar Degradation in Lignocellulosic Hydrolysate Utilization

Xylose and ribose metabolism was higher in the fecal microbiome of the Lantang pig (*P* < 0.05), but the metabolic capacities of fructose and mannose were higher in the fecal microbiome of Duroc pigs (*P* < 0.05). There were no differences in other monosaccharides between the two fecal microbiomes (*P* > 0.05) (Supplementary Figure S4 and Supplementary Table S1).

#### Microbial Central Metabolism in Lignocellulosic Hydrolysate Utilization

The abundance of the gene coded fumarate reductase (k00244) involved in the TCA pathway was higher in the fecal microbiome of the Lantang pig than in the Duroc pig (*P* < 0.05), so the potential effectiveness of TCA pathway was better in the fecal microbiome of the Lantang pig than in the Duroc pig (*P* < 0.05), but there were no significant differences in glycolysis or pentose phosphate pathways between the two microbiomes (*P* > 0.05) (Supplementary Figure S5 and Supplementary Table S1).

#### Microbial Hydrogen Sink System in Lignocellulosic Hydrolysate Utilization

**FIGURE 2:**
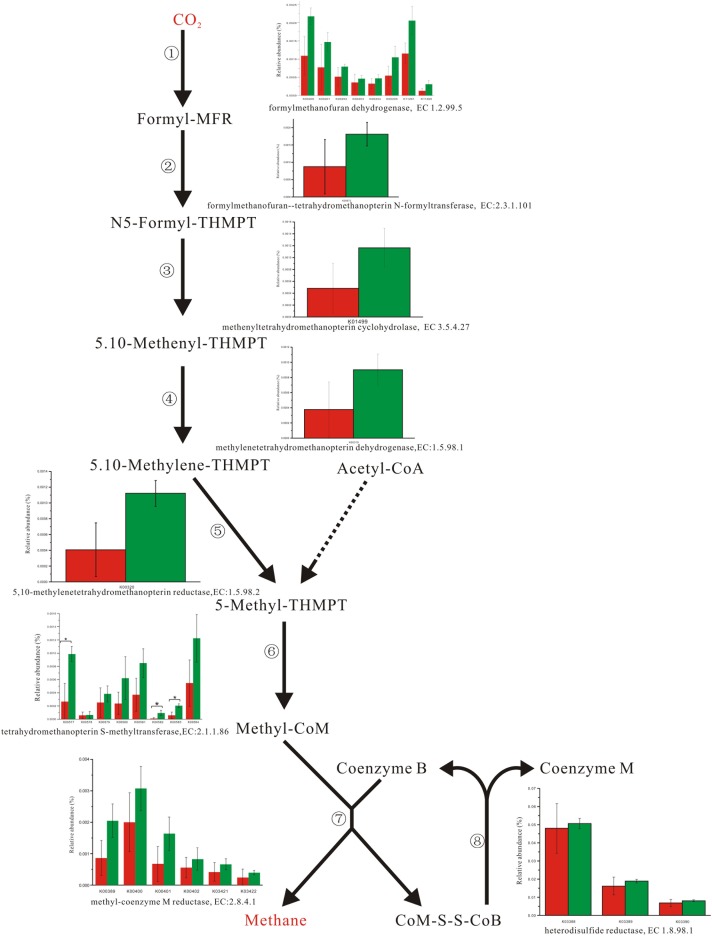
Comparison of abundance for gene function involved in methane metabolism. Red and Green represent the Duroc group (DR) and the Lantang group (LT), respectively. Asterisk (^∗^) denotes *P* < 0.05. The dashed line represents Minor metabolic pathway in methane metabolism from KEGG reference pathway.

**FIGURE 3:**
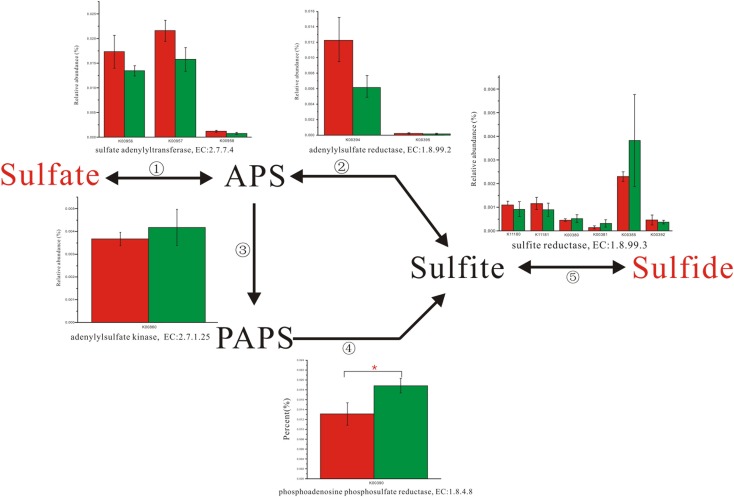
Comparison of abundance for gene function involved in hydrogen sulfide metabolism. Red and Green represent the Duroc group (DR) and the Lantang group (LT), respectively. Asterisk (^∗^) denotes *P* < 0.05.

Enzyme abundance in the hydrogen sink pathway, which involves the production of methane (**Figure 2**) and hydrogen sulfide (**Figure 3**), was also compared between the two fecal microbiomes. Gene abundance in the hydrogen sink pathway was higher in the fecal microbiome from Lantang pigs relative to Duroc pigs (*P* < 0.05). However, there was no distinct difference in the amount of hydrogen that would be abated by the production of acetic acid (*P* > 0.05) (Supplementary Figure S6).

## Discussion

The main goal of this study was to investigate the microbiological potential for dietary fiber utilization between the Chinese native Lantang and the commercial Duroc pigs by comparing their structural and functional metagenomes of their fecal microbiomes. Due to continuous changes in the composition of fecal microbes during each progressive developmental stage, it is important to select the appropriate life stage of the host animal to accurately compare the microbiological potential for fiber utilization. Previous studies have shown that the GIT microbiota in pigs achieves a relatively stable condition at 6 months of age and that the majority of microbes in the feces are shared with the large intestine (Kim et al., 2011; Zhao et al., 2015). In addition, a recent study indicated the relative abundance of ABC transporters and the phosphotransferase system from female pig gut microbiome were higher than male pig (Xiao et al., 2016). ABC transporters and the phosphotransferase system were mainly responsible for the sugar transport system and played an important role in fiber utilization (Sauvageot et al., 2017). So we predicted that the potential for utilization of fiber by female pigs were better than that of male pigs. Thus, the fecal microbiome from 150-day-old female pigs was used to study the utilization capacity of dietary fiber among the two pig breeds.

Several studies have reported that host genetics shapes the gut microbiota in mice and in humans (Rajilić-Stojanović et al., 2007; Turnbaugh et al., 2009b; Campbell et al., 2012; Hildebrand et al., 2013). Previous study that investigated the similarities and differences in the pig fecal microbiota among three 15-week-old pure bred pig lines, namely Duroc, Landrace, and Yorkshire breeds (Pajarillo et al., 2014), showed that the three breeds shared similar GIT bacterial community and with distinct compositions. In the present study, we found that 53 low-abundance genera were distinct between the two pig breeds, with 11 genera being higher in the fecal microbiome of Duroc pigs while the remaining 42 genera were higher in the Lantang fecal microbiome. Research has shown that bacteria from some of the above mentioned 42 genera are related to fiber degradation, including the amount of digested NSP, xylose, and dietary fiber in ileal digesta, which are positively correlated to the abundance of *Bacteroides– Prevotella– Porphyromonas* (Ivarsson et al., 2014). Additionally, *Alistipes putredinis* can degrade fiber and glucosinolates (Li et al., 2009), and *Alistipes finegoldii* is involved in glycan metabolism (Nihira et al., 2013). In the present study, the abundance of *Porphyromonas* and *Alistipes* was higher in Lantang pigs. In humans, De Filippo et al. (2010) showed that African children who consumed a high-fiber diet had significant enrichment of the genus *Cytophaga* in their fecal samples compared with European children consuming a modern Western diet. The above study showed that two of the most striking characteristics of *Cytophaga hutchinsonii* are its rapid gliding motility over surfaces and its contact-dependent digestion of crystalline cellulose. Gliding may help to facilitate cellulose digestion, since gliding cells align themselves with cellulose fibers and digest them as they move along the fibers. (Xie et al., 2007). In the present study, the abundance of *Cytophaga* was higher in the fecal microbiome of Lantang pigs, which are usually fed with diet consisting of large proportion of fibrous agricultural byproducts. All pigs in this study were housed in individual pens and fed the same diet without any antibiotic treatment. Therefore, any differences in the GIT bacterial communities should be breed differences, and the higher fiber digestion capacity demonstrated by the Lantang pigs could be the higher abundance of bacteria associated with fiber digestion in the large intestine.

To compare the fiber-degrading capabilities between the two pig breeds, microbiome gene-centric metagenomics datasets were constructed and annotation focused on identifying putative carbohydrate-active enzyme genes, which are necessary for fiber degradation in the extracellular environment. The GIT bacteria produce a vast amount of CAZymes to digest dietary fibers into metabolisable monosaccharides and disaccharides (Tasse et al., 2010; El Kaoutari et al., 2013). Several metagenomics studies focusing on the GIT microbiome aimed to determine the diversity of CAZymes revealed that the human GIT microbiome is a surprisingly rich source of carbohydrate-active enzymes (Gill et al., 2006; Turnbaugh et al., 2009a). Glycoside hydrolases (GHs) are one of the dominant enzyme classes in the GIT microbiota. These enzymes mainly hydrolyse the glycosidic linkage of glycosides and play a crucial role in the digestion of complex carbohydrates, such as those found in the plant cell wall. GHs may contain single or multiple catalytic GHs, together with single or multiple non-catalytic carbohydrate-binding modules (CBMs). In the present study, we found that the abundance of GH and CBM genes was higher in the fecal microbiome of Lantang pigs than in Duroc pigs, suggesting that Lantang pigs have a higher efficacy of dietary fiber utilization relative to Duroc pigs (Cheng et al., 2017).

Additionally, the gene numbers of debranching enzymes and oligosaccharide-degrading enzymes were higher in the fecal microbiome of from Lantang pigs. Debranching enzymes mainly eliminate the branch chain of hemicellulose. In the debranching enzymes system, the most frequently occurring GH families in the two fecal microbiomes were GH78 and GH51, which mainly degrade the a-1,2 and a-1,3-arabinofuranosidic bonds of the branch chain from arabinose. In the oligosaccharide-degrading enzyme system, GH43 was predominant in Lantang pigs, but the difference was not significant. This was followed by GH3, GH2, and GH29. This finding suggests that the microbiome of Lantang pigs may have a higher capacity for hemicellulose degradation.

Dietary fibers are degraded into monosaccharides, disaccharides and oligosaccharides in the extracellular environment and are transported into bacterial cells by specific transporter proteins. The role of transporters in sugar uptake is central to a microorganism’s, ability to utilize lignocellulosic substrates (Jojima et al., 2010). The results of our study show that the transport ability of xylose, arabinose, and ribose in the fecal microbiome of Lantang pigs was dominant in the KEGG database analysis, demonstrating that Lantang pigs make highly efficient use of dietary fiber with a high xylose and arabinose content.

The process of intracellular carbohydrate degradation entering is divided into two parts: monosaccharides (or disaccharides) and central (glycolysis, pentose phosphate pathway, and TCA cycle) metabolism. The end products of degradation are volatile fatty acids(VFAs), CO_2_, and H_2_. The results of our KEGG analysis of the fecal microbiome showed a higher metabolic capacity of xylose, ribose, and fucose but a lower capacity for fructose and mannose for Lantang pigs compared to Duroc pigs. We hypothesized that the difference was mainly due to dietary adaptation of the microbial system. A recent study in humans showed that the microbiome from Chinese subjects contained more enzymes responsible for breaking down di- and mono-saccharides such as maltose, trehalose, fructose, mannose and galactose than the microbiome from American subjects (Li et al., 2014), which could be because rice, which is easily converted into simple sugars, is the main carbohydrate source for many Chinese individuals (Gonlachanvit, 2010).

Generally, the products of pentose and hexose metabolism enter the pentose phosphate pathway and glycolysis, respectively, before entering the TCA cycle. In the present study, the potential capacity of the TCA cycle was significantly higher in the fecal microbiome of Lantang pigs than Duroc pigs. Due to the energy produced by the TCA cycle, this could accelerate bacterial cell growth.

In the pig GIT, dietary components, including fiber, that reach the colon are fermented principally to VFAs, H_2_, and CO_2_. The accumulation of H_2_ leads to reduced fermentation and/or less energy-efficient fermentation. Thus, the microbial disposal of H_2_, generated during anaerobic fermentation in the pig large intestine, is critical for optimal functioning of this ecosystem. In the absence of H_2_-consuming organisms, the H_2_ partial pressure rapidly reaches a level that thermodynamically restricts fermentation. There are three major groups of H_2_-consuming microorganisms in the human colon, namely methanogens, sulfate-reducing bacteria (SRB), and acetogens (Nakamura et al., 2010), but few studies have reported on the H_2_ sink in the pig GIT. Based on the KEGG database analysis, we detected three complete H_2_ sink pathways in pigs and found that tetrahydromethanopterin S-methyltransferase and phosphoadenosine phosphosulfate reductase, which are involved in the methane and hydrogen sulfide production pathways, respectively, were higher in the fecal microbiome from Lantang pigs than Duroc pigs. However, the potential dominance of the H_2_ sink on acetate production between the two breeds was the same. Thus, the potential dominance of the H_2_ sink in Lantang pigs may provide an advantage over Duroc pigs for fiber degradation.

## Conclusion

Metagenomics is a powerful tool that can be used to describe the genetic potential of the microorganisms present in a given environment. From the results of the present study, we conclude that the capacity of fiber utilization is higher for native Lantang pigs compared to Duroc pigs. The high fiber utilization efficiency in Lantang pigs is due to their inherent microbiological adaptations to high-fiber diets, which include (i) the structural differences of the fecal microbial community with several genera associated with fiber utilization; (ii) the potential of putative functional genes and the amount of genes encoding debranching enzymes, oligosaccharide-degrading enzymes, and lignocellulose bonding modules, which contribute to extracellular fiber degradation; (iii) the transport capacity and intracellular metabolism of xylose, L-arabinose, and ribose by the fecal microbiome; and (iv) the potential capacity in the TCA cycle pathway and the potential of H_2_ sinks in the production of methane and hydrogen sulfide.

## Author Contributions

PC, YaW, JL, YiW, and XL designed the study. PC collected the samples, performed the sequence analysis and annotation, performed the metagenomic analysis, and wrote the manuscript. YaW, JL, YiW, AW, and XL revised the manuscript. All authors read and approved the final manuscript.

## Conflict of Interest Statement

The authors declare that the research was conducted in the absence of any commercial or financial relationships that could be construed as a potential conflict of interest.
