# Supplementary material for: Exploratory Analysis of the Microbiological Potential for Efficient Utilization of Fiber Between Lantang and Duroc Pigs
Source: Front Microbiol. 2018 Jun 22;9:1342. doi: 10.3389/fmicb.2018.01342 (PMC6023970; doi:10.3389/fmicb.2018.01342)
Supplement: Supplementary file 1 [file Data_Sheet_1.DOCX]

Figure S1 Rarefaction curves for all samples in the genus level
