# Supplementary figures and images for: Exploratory Analysis of the Microbiological Potential for Efficient Utilization of Fiber Between Lantang and Duroc Pigs
Source: Front Microbiol. 2018 Jun 22;9:1342. doi: 10.3389/fmicb.2018.01342 (PMC6023970; doi:10.3389/fmicb.2018.01342)

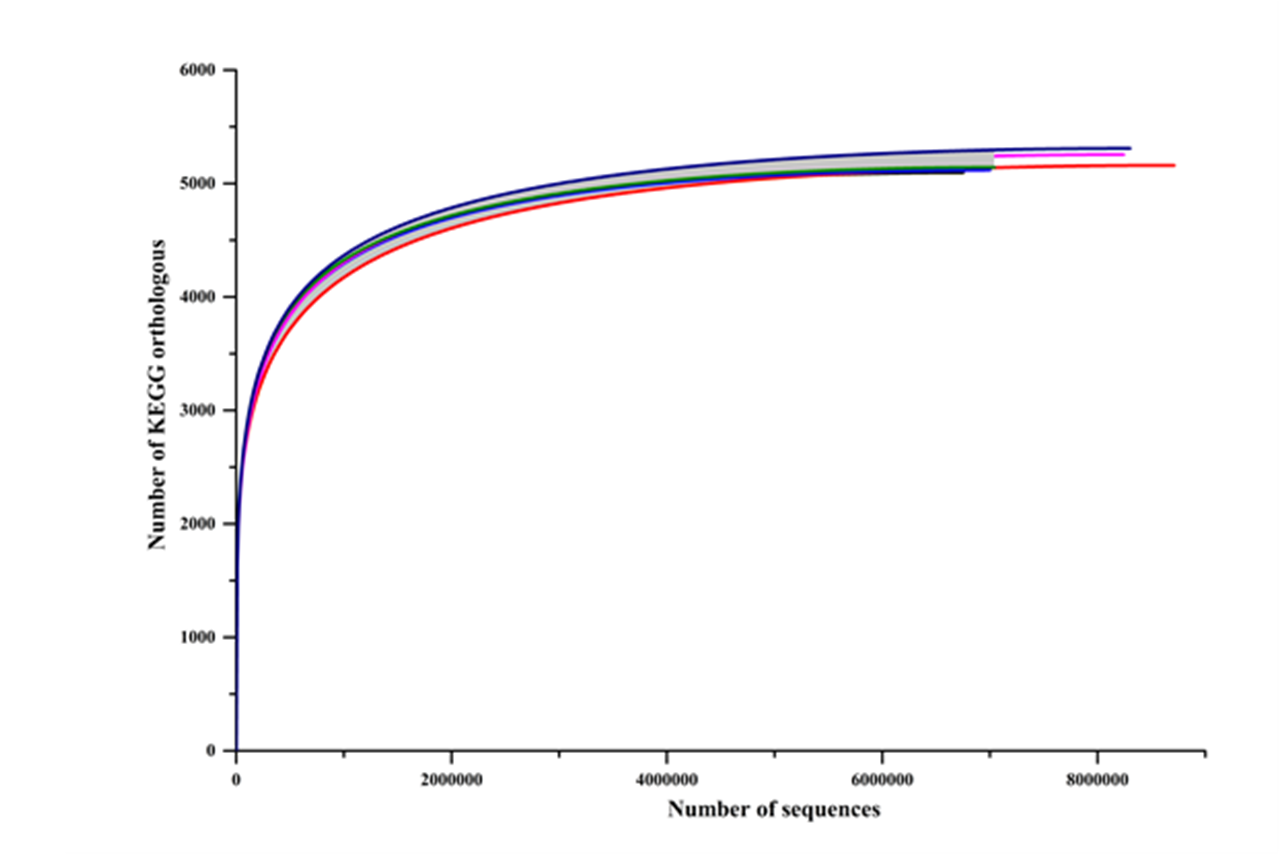


#### Figure S2. KEGG Functional accumulation curves for all samples

Supplement: Supplementary file 2 [file Data_Sheet_2.DOCX]
