# Supplementary material for: Exploratory Analysis of the Microbiological Potential for Efficient Utilization of Fiber Between Lantang and Duroc Pigs
Source: Front Microbiol. 2018 Jun 22;9:1342. doi: 10.3389/fmicb.2018.01342 (PMC6023970; doi:10.3389/fmicb.2018.01342)
Supplement: Supplementary file 3 [file Data_Sheet_3.DOCX]

**Figure S3. Comparison of sugar transporters in the faecal bacterial community from two pig breeds.** Red and Green represent the Drouc group (DR) and the Lantang group (LT), respectively. Asterisk denoted *P*<0.05, **indicated *P*<0.01; * indicated P<0.05.

(A)


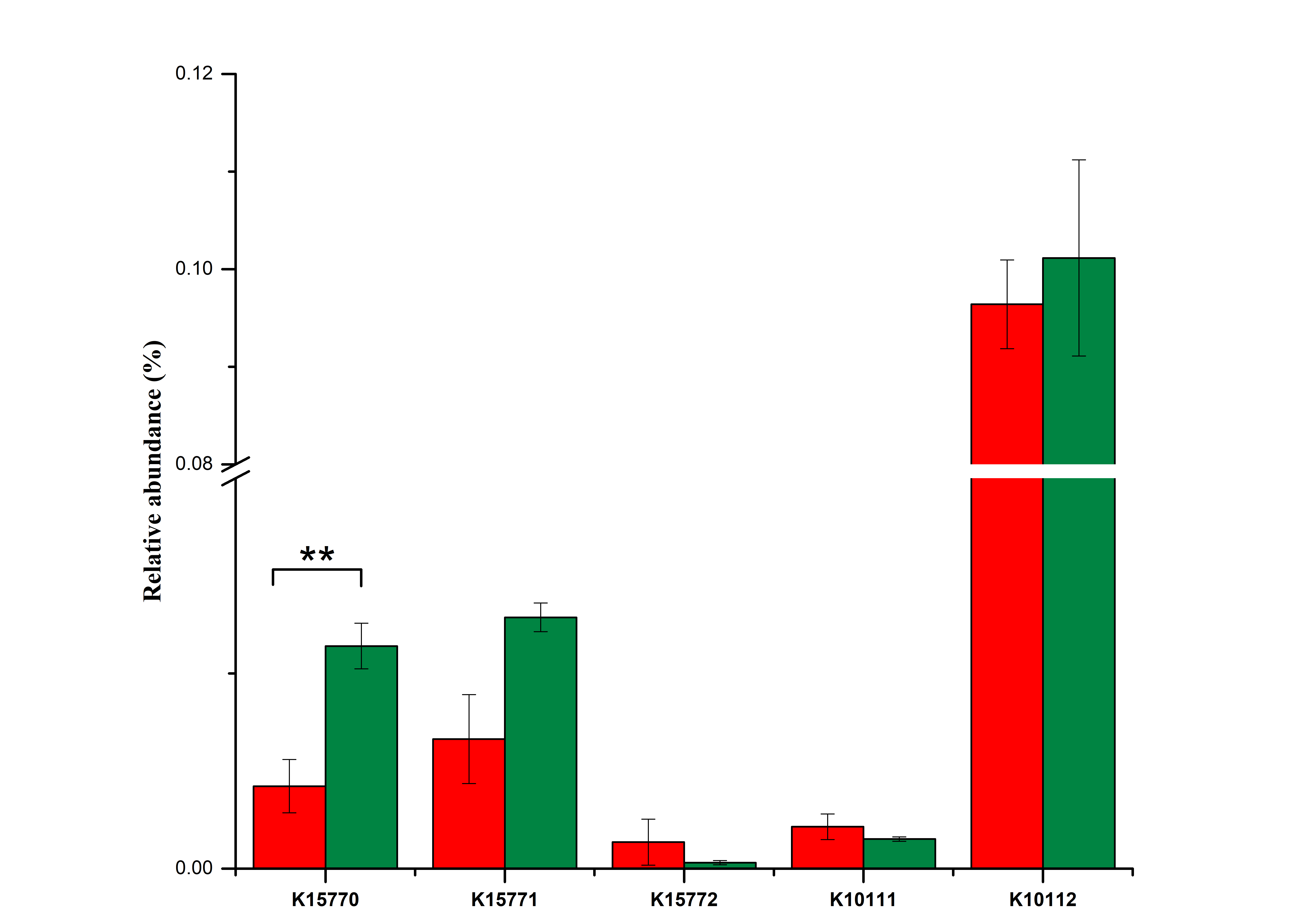


Comparison of glycan/maltooligosaccharide transporters in the faecal bacterial community from two pig breeds

(B)





Comparison of lactose/L- arabinose transporters in the faecal bacterial community from two pig breeds

(C)


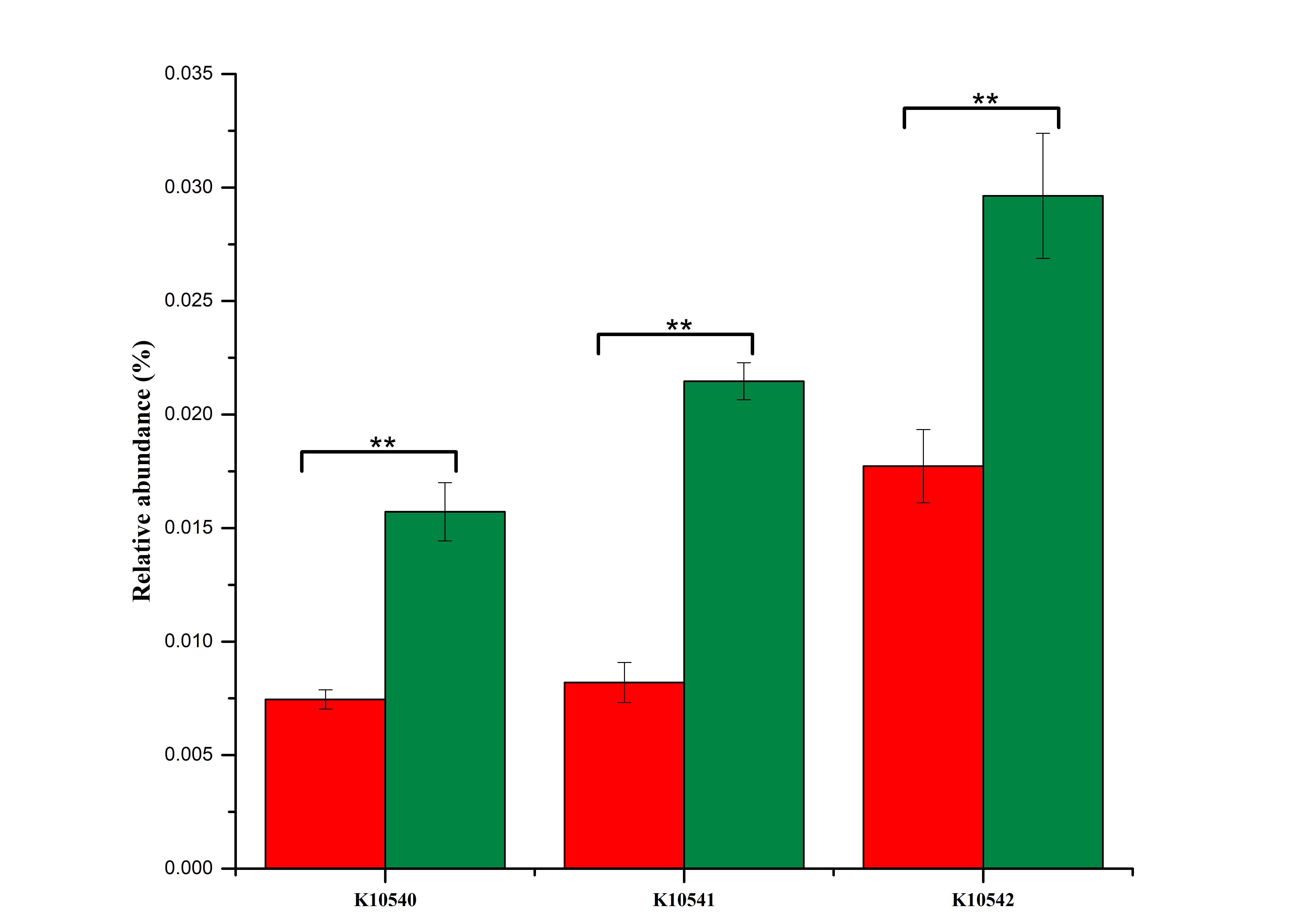


Comparison of methy-lgalactoside transporters in the faecal bacterial community from two pig breeds

(D)





Comparison of D-xylose transporters in the faecal bacterial community from two pig breeds

(E)





Comparison of ribose transporters in the faecal bacterial community from two pig breeds

(F)





Comparison of Raffinose/stachyose/melibiose transporters in the faecal bacterial community from two pig breeds
