# Supplementary material for: Exploratory Analysis of the Microbiological Potential for Efficient Utilization of Fiber Between Lantang and Duroc Pigs
Source: Front Microbiol. 2018 Jun 22;9:1342. doi: 10.3389/fmicb.2018.01342 (PMC6023970; doi:10.3389/fmicb.2018.01342)
Supplement: Supplementary file 4 [file Data_Sheet_4.DOCX]

**Figure S4: Bacterial simple sugar degradation in lignocellulosic hydrolysate utilization.** Red and Green represent the Drouc group (DR) and the Lantang group (LT), respectively. Asterisk denoted P<0.05, **indicated P<0.01; * indicated P<0.05.


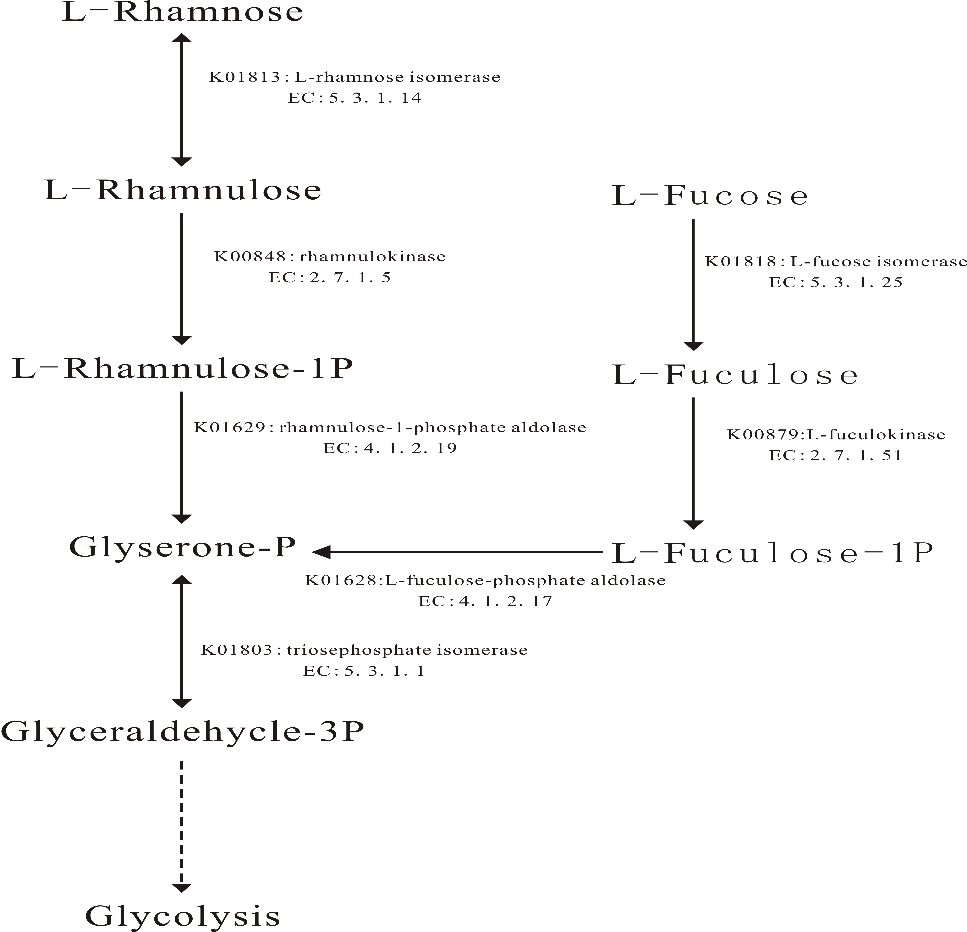


Degradation process of rhamnose and fucose


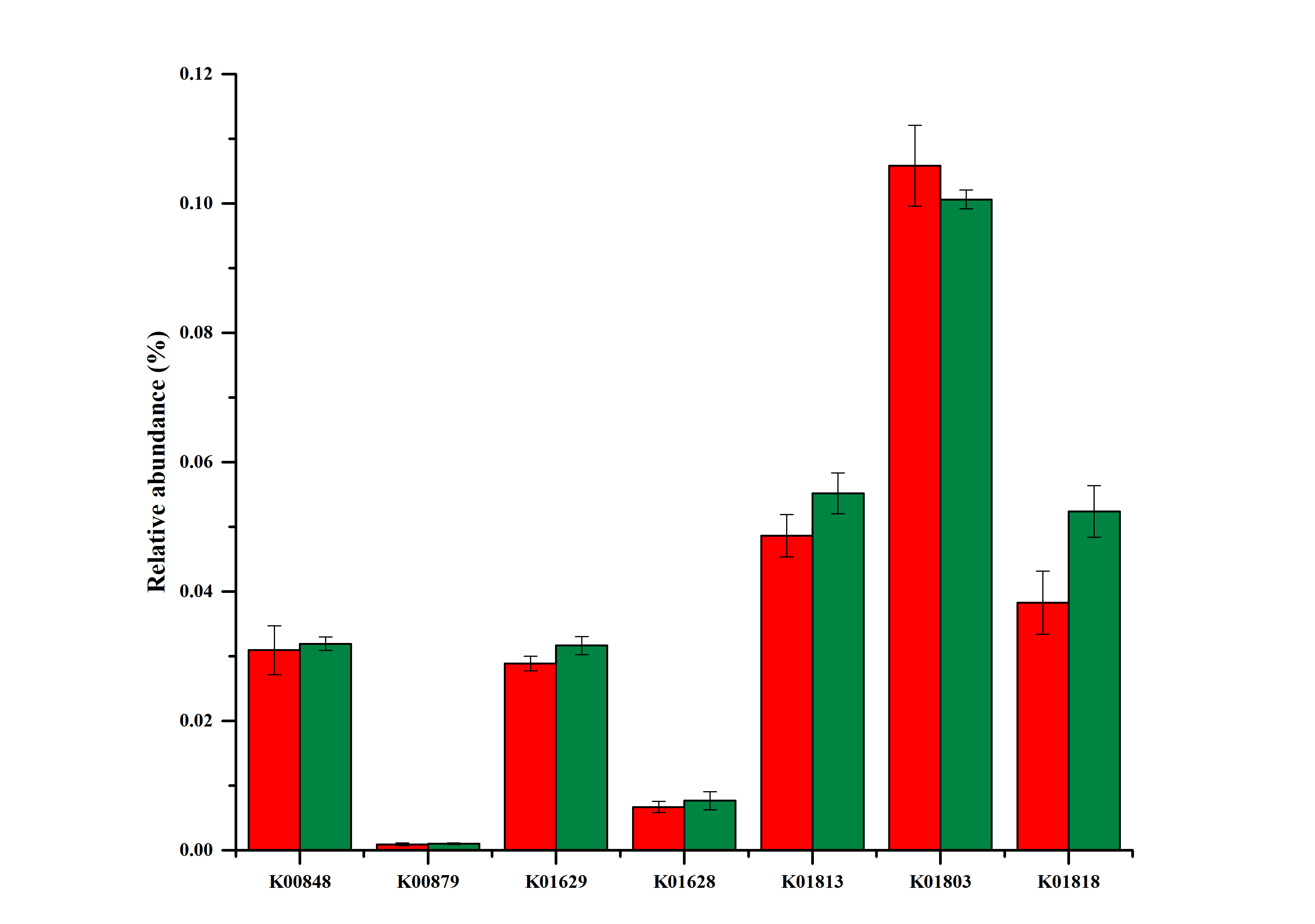


Comparison of abundance for function gene degrading rhamnose and fucose among the two breed pigs metagenome


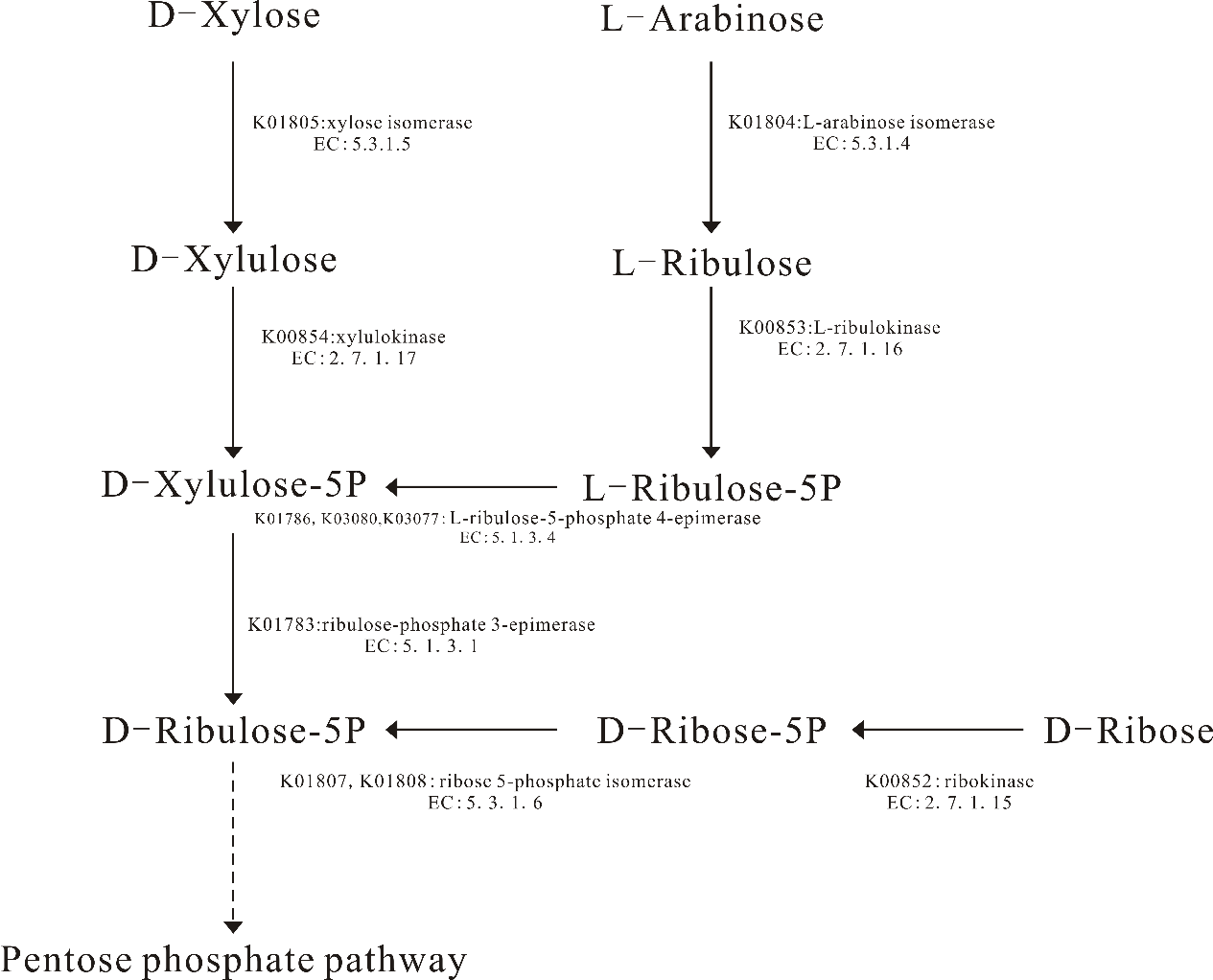


Degradation process of pentose


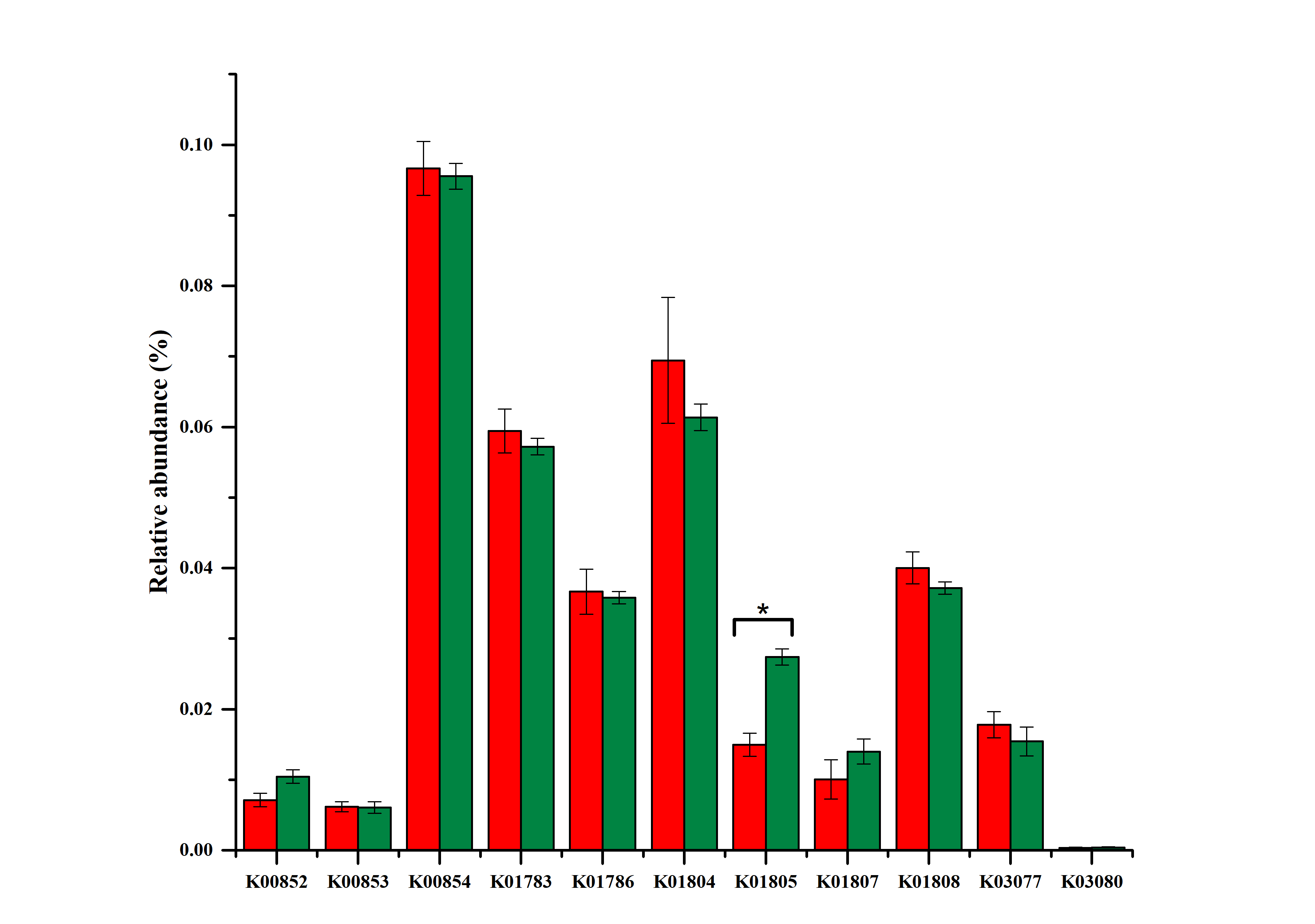


Comparison of abundance for function gene degrading pentose among the two breed pigs metagenome


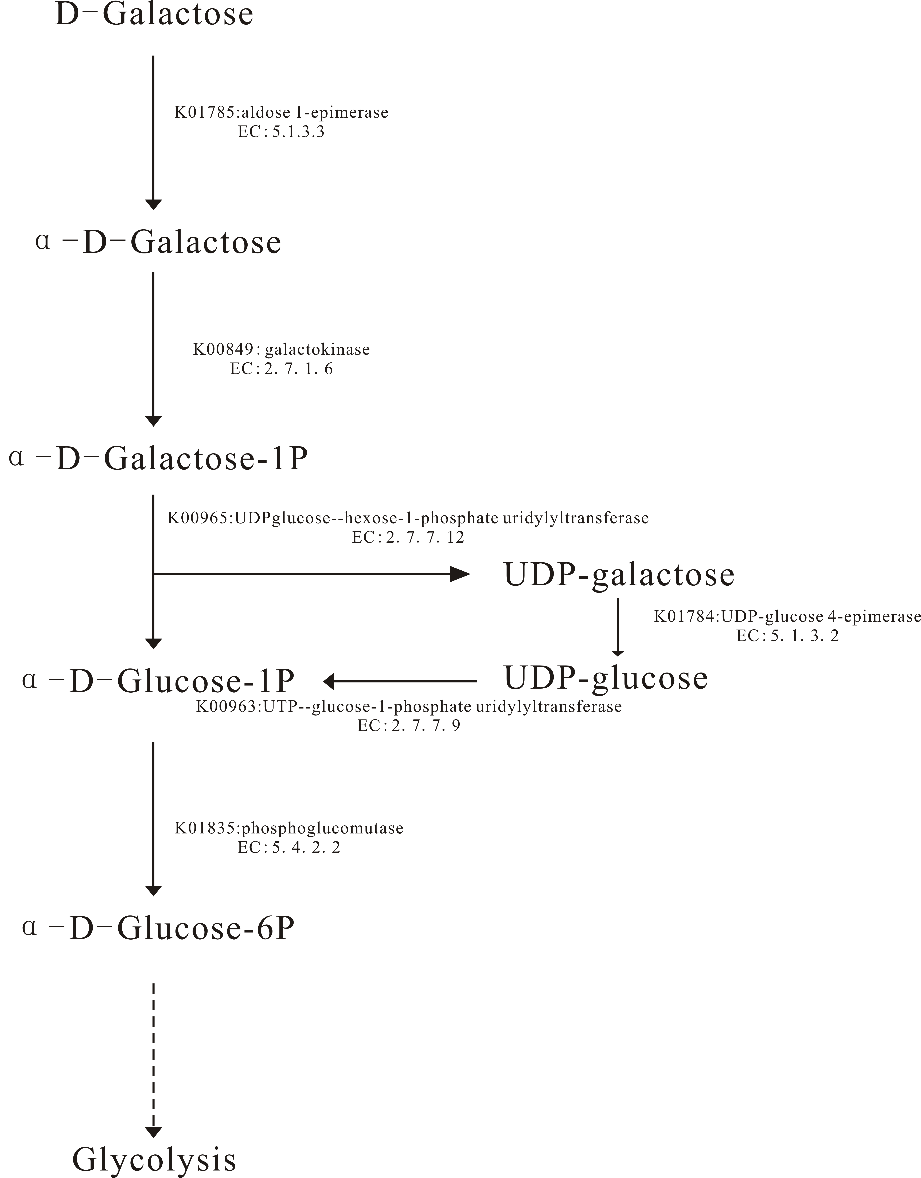


Degradation process of galactose





Comparison of abundance for function gene degrading galactose among the two breed pigs metagenome


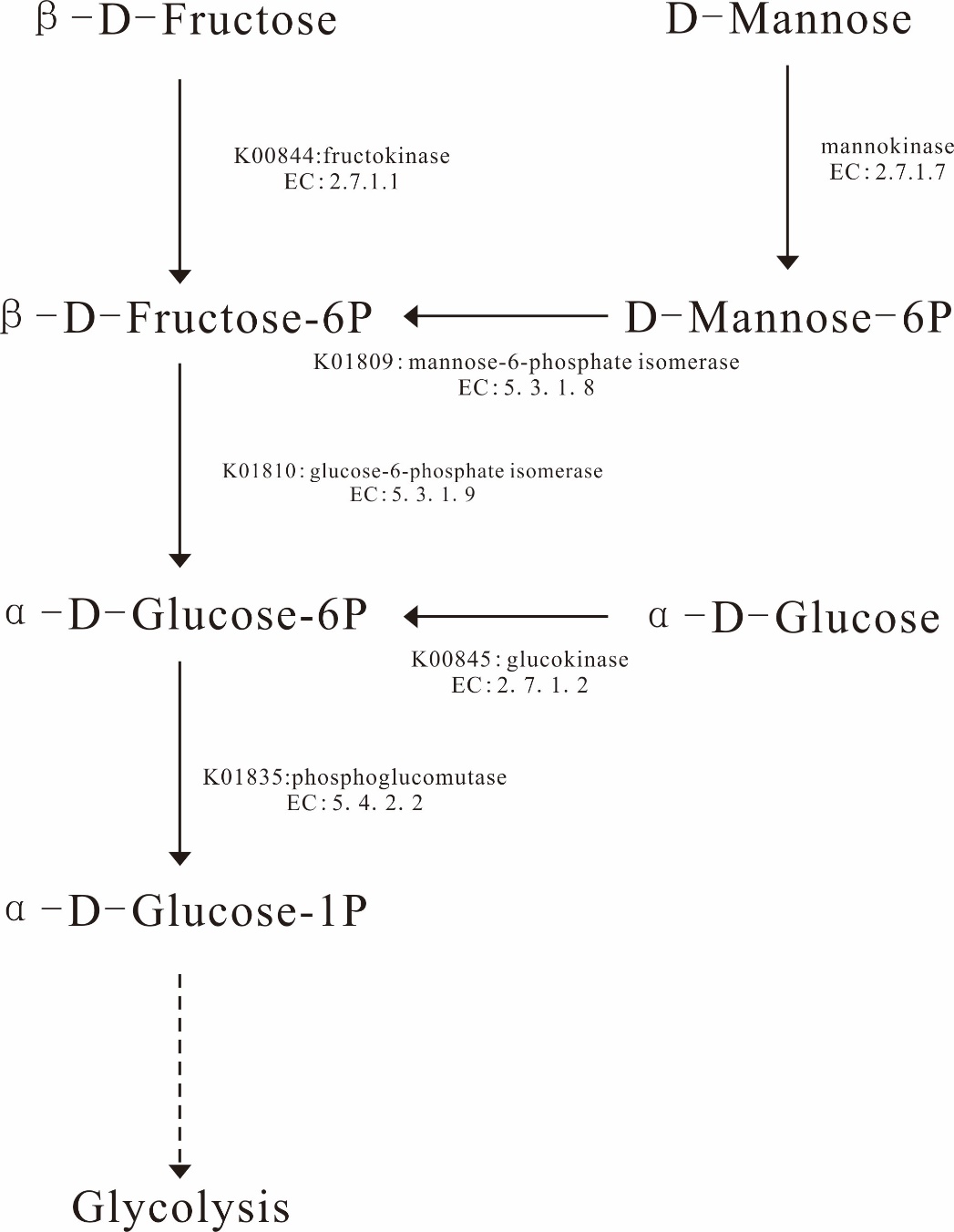


Degradation process of fructose, mannose and glucose


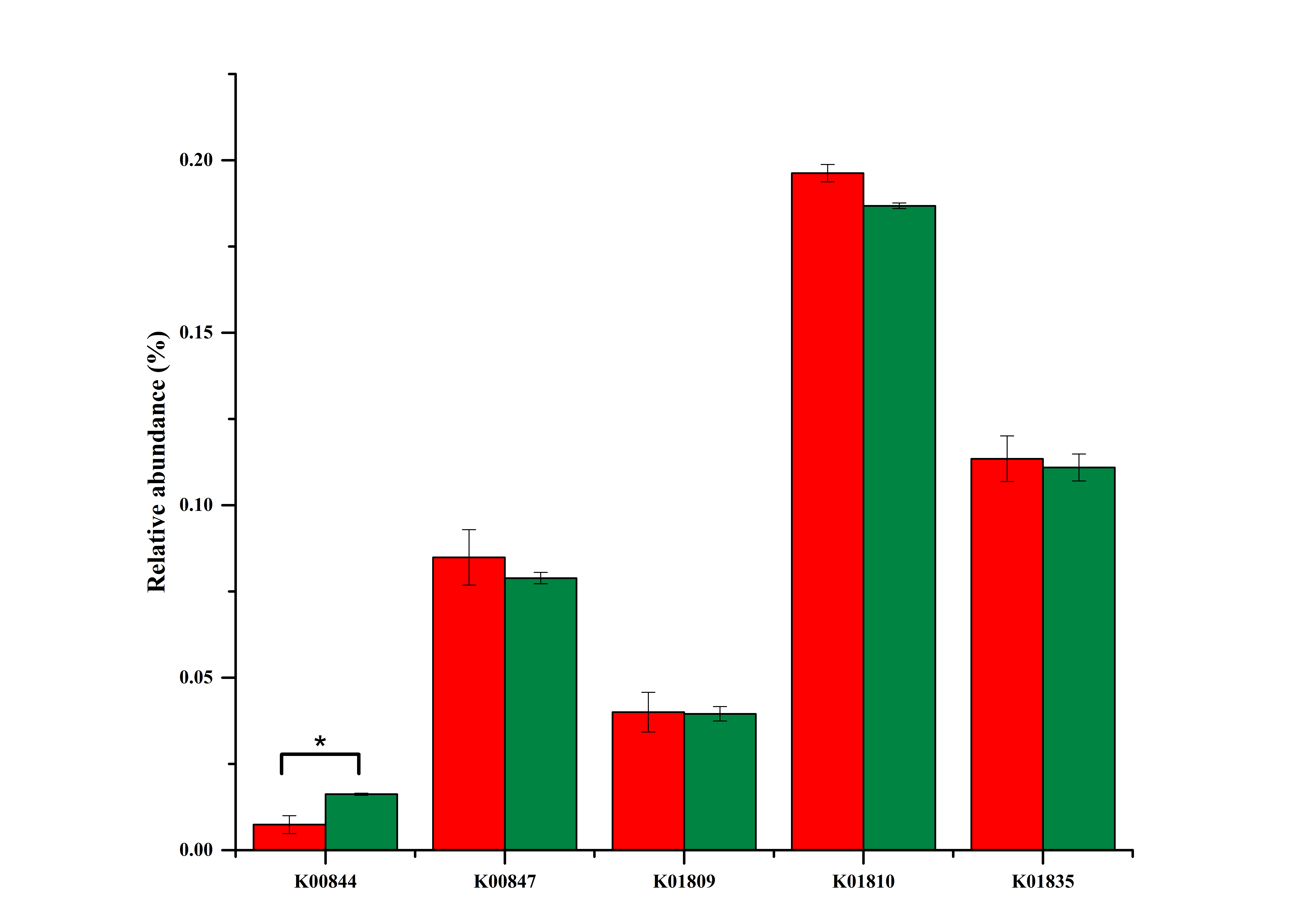


Comparison of abundance for function gene degrading fructose, mannose and glucose among the two breed pigs metagenome
