# Supplementary material for: Exploratory Analysis of the Microbiological Potential for Efficient Utilization of Fiber Between Lantang and Duroc Pigs
Source: Front Microbiol. 2018 Jun 22;9:1342. doi: 10.3389/fmicb.2018.01342 (PMC6023970; doi:10.3389/fmicb.2018.01342)
Supplement: Supplementary file 5 [file Data_Sheet_5.DOCX]

**Figure S5. Microbial Central metabolism in lignocellulosic hydrolysate utilization.** Red and Green represent the Drouc group (DR) and the Lantang group (LT), respectively. Asterisk denoted P<0.05, **indicated P<0.01; * indicated P<0.05.

**

**

Comparison of abundance for function gene involved in pentose phosphate pathway among the two breed pigs metagenome





Comparison of abundance for function gene involved in Core Module of glycolysis among the two breed pigs metagenome





Comparative of abundance for function gene involved in TCA among the two breed pigs metagenome
