# Supplementary material for: Exploratory Analysis of the Microbiological Potential for Efficient Utilization of Fiber Between Lantang and Duroc Pigs
Source: Front Microbiol. 2018 Jun 22;9:1342. doi: 10.3389/fmicb.2018.01342 (PMC6023970; doi:10.3389/fmicb.2018.01342)

**Figure S6. Comparison of abundance for gene function involved in the process of producing acetate.** Red and Green represent the Drouc group (DR) and the Lantang group (LT), respectively. No asterisk indicates that the difference is not significant.


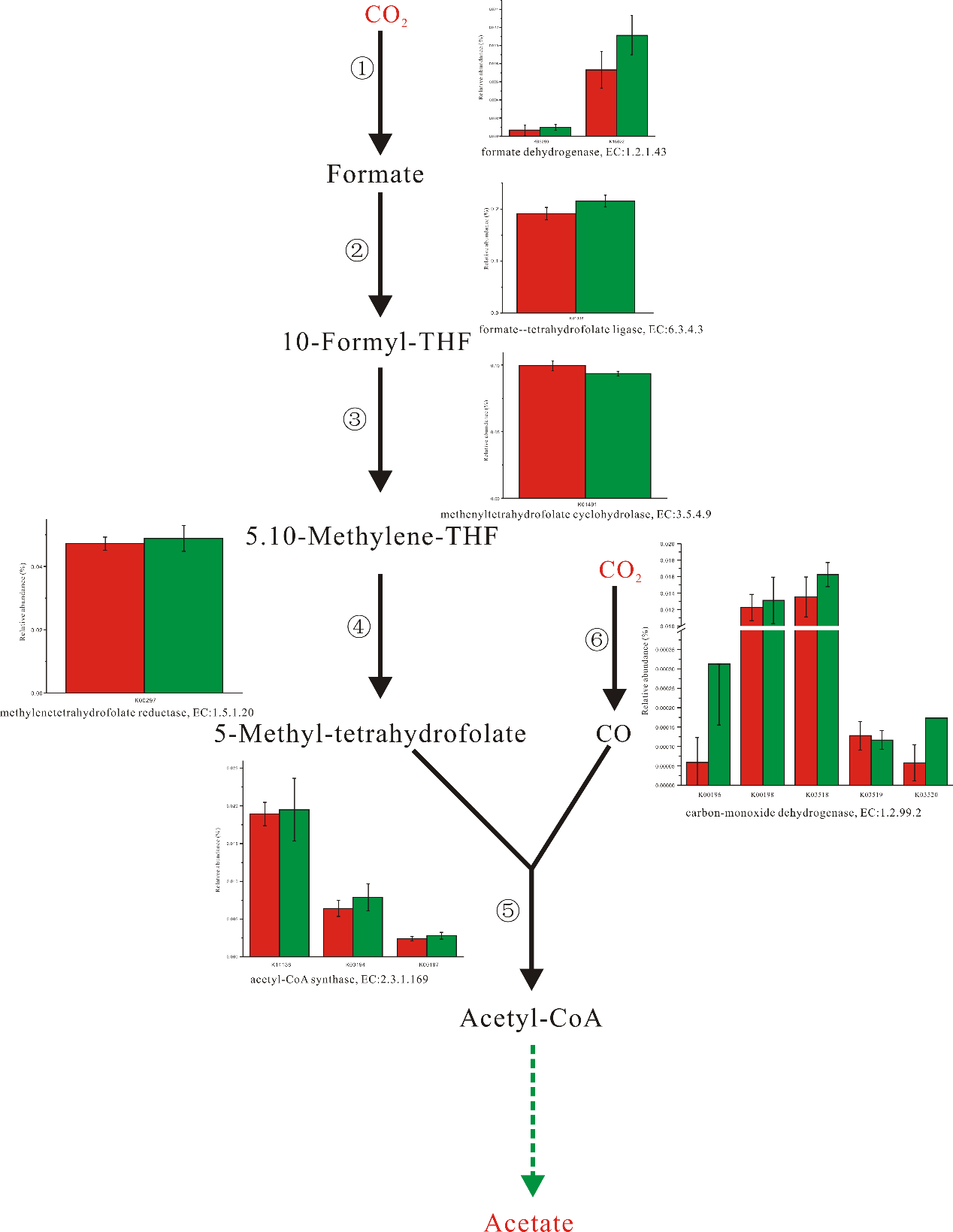

Supplement: Supplementary file 6 [file Data_Sheet_6.docx]
